# Artificial intelligence for quantifying immune infiltrates interacting with stroma in colorectal cancer

**DOI:** 10.1186/s12967-022-03666-3

**Published:** 2022-10-04

**Authors:** Jing Yang, Huifen Ye, Xinjuan Fan, Yajun Li, Xiaomei Wu, Minning Zhao, Qingru Hu, Yunrui Ye, Lin Wu, Zhenhui Li, Xueli Zhang, Changhong Liang, Yingyi Wang, Yao Xu, Qian Li, Su Yao, Dingyun You, Ke Zhao, Zaiyi Liu

**Affiliations:** 1 Guangdong Provincial Key Laboratory of Artificial Intelligence in Medical Image Analysis and Application, Guangdong Provincial People’s Hospital, Guangzhou, China; 2 Department of Cardiology, Sun Yat-Sen Memorial Hospital, Sun Yat-Sen University, Guangzhou, China; 3 Department of Radiology, Guangdong Provincial People’s Hospital, Guangdong Academy of Medical Sciences, 106 Zhongshan Er Road, Guangzhou, 510080 China; 4 The Second School of Clinical Medicine, Southern Medical University, Guangzhou, China; 5 Department of Pathology, The Sixth Affiliated Hospital of Sun Yat-Sen University, Guangzhou, China; 6 Department of Radiology, The Sixth Affiliated Hospital of Sun Yat-Sen University, Guangzhou, China; 7 Department of Pathology, The Third Affiliated Hospital of Kunming Medical University, Yunnan Cancer Hospital, Yunnan Cancer Center, Kunming, China; 8 Department of Radiology, The Third Affiliated Hospital of Kunming Medical University, Yunnan Cancer Hospital, Yunnan Cancer Center, Kunming, China; 9 Department of Ophthalmology, Guangdong Eye Institute, Guangdong Provincial People’s Hospital, Guangdong Academy of Medical Sciences, Guangzhou, China; 10 Department of Radiology, Zhuhai People’s Hospital, Zhuhai Hospital Affiliated With Jinan University, Zhuhai, China; 11 School of Medicine, South China University of Technology, Guangzhou, China; 12 Department of Pathology, Guangdong Provincial People’s Hospital, Guangdong Academy of Medical Sciences, 106 Zhongshan Er Road, Guangzhou, 510080 China; 13 School of Public Health, Kunming Medical University, 191 West Renmin Road, Kunming, 650500 China; 14 Guangdong Cardiovascular Institute, Guangdong Provincial People’s Hospital, Guangdong Academy of Medical Sciences, Guangzhou, China

**Keywords:** Deep learning, Whole-slide images, Deep-immune score, Colorectal cancer, Digital pathology

## Abstract

**Background:**

We proposed an artificial intelligence-based immune index, Deep-immune score, quantifying the infiltration of immune cells interacting with the tumor stroma in hematoxylin and eosin-stained whole-slide images of colorectal cancer.

**Methods:**

A total of 1010 colorectal cancer patients from three centers were enrolled in this retrospective study, divided into a primary (N = 544) and a validation cohort (N = 466). We proposed the Deep-immune score, which reflected both tumor stroma proportion and the infiltration of immune cells in the stroma region. We further analyzed the correlation between the score and CD3^+^ T cells density in the stroma region using immunohistochemistry-stained whole-slide images. Survival analysis was performed using the Cox proportional hazard model, and the endpoint of the event was the overall survival.

**Result:**

Patients were classified into 4-level score groups (score 1–4). A high Deep-immune score was associated with a high level of CD3^+^ T cells infiltration in the stroma region. In the primary cohort, survival analysis showed a significant difference in 5-year survival rates between score 4 and score 1 groups: 87.4% vs. 58.2% (Hazard ratio for score 4 vs. score 1 0.27, 95% confidence interval 0.15–0.48, P < 0.001). Similar trends were observed in the validation cohort (89.8% vs. 67.0%; 0.31, 0.15–0.62, < 0.001). Stratified analysis showed that the Deep-immune score could distinguish high-risk and low-risk patients in stage II colorectal cancer (P = 0.018).

**Conclusion:**

The proposed Deep-immune score quantified by artificial intelligence can reflect the immune status of patients with colorectal cancer and is associate with favorable survival. This digital pathology-based finding might advocate change in risk stratification and consequent precision medicine.

**Supplementary Information:**

The online version contains supplementary material available at 10.1186/s12967-022-03666-3.

## Background

The immune system was an essential component of the tumor microenvironment (TME) in colorectal cancer (CRC) [1]. It played a central role in tumorigenesis and progression, affecting the treatment and prognosis of CRC [2]. The need to go beyond the tumor-node-metastasis (TNM) staging system has been addressed by detecting the tumor immune microenvironment (TIME), which was affected by the type, density, and location of tumor-infiltrating lymphocytes (TILs) [3–5]. The Immunoscore®, calculated based on the density of CD3^+^ and CD8^+^ T cells in the tumor core and invasive margin, has been shown to provide superior prognosis to TNM stage in CRC [4, 6]. However, most of the current immune scores needed to be stained with immunohistochemistry (IHC) or other special staining (such as multiplexed immunofluorescence), which is not commonly used in clinical practice, hindering their widespread clinical application.

In most cases, high-quality hematoxylin and eosin (HE)-stained slide was sufficient to confirm the diagnosis [7]. Immune-related features within HE-stained whole-slide images (WSIs), such as TILs and Crohn-like lymphoid reaction (CLR), can be quantified using deep learning. HE-stained slides also have immunological information, with a larger sample size and lower cost. Deep learning has recently entered the field of computational pathology and shows excellent promise for task automation [8]. Deep learning with digital pathology has been successfully applied to breast, prostate, lung, and CRC [9–15]. The tissue-level components of CRC, such as tumor-stroma ratio (TSR) and CLR [16, 17], can be quantified using deep learning. Therefore, the artificial intelligence (AI)-based method has the potential to quantify the tissue composition and immune status using HE-stained WSIs.

The spatial distribution of TILs was important for CRC prognosis. TILs can interact with the tumor through direct contact or cytokine signaling to produce tumor-killing immune cells for protection of the organism, mainly in the interstitial region [18]. The immune cells in the stroma are produced by the surrounding lymphoid follicles or migrate from the blood to the tumor area. As the stroma increases, the number of immune cells in the stroma decreases and the anti-tumor effect decreases [16]. In addition, the distribution of immune cells in the stroma impacted the prognosis of CRC [19]. We hypothesized, therefore, that a comprehensive consideration of the stroma proportion and the immune cell infiltration in stroma would further refine the prognostic stratification of CRC patients.

The aim of this study was two-fold. First, we proposed a deep learning-based immune index, the Deep-immune score, quantifying immune infiltration interaction with the stroma in HE-stained WSIs. A further investigation of its prognostic value is performed in CRC patients from three centers.

## Methods

### Patients

Our study recruited patients with histologically confirmed stage I–III CRC who had undergone surgery with the intent of curing their cancer, and had paraffin-embedded tumor samples available (Additional file 1: Fig. S1). The primary cohort consists of patients from Guangdong Provincial People's Hospital (from Apr 2008 to Jun 2016), and the validation cohort includes patients from Yunnan Cancer Hospital (from Dec 2012 to Apr 2015) and The Sixth Affiliated Hospital of Sun Yat-sen University (from Jan 2013 to Oct 2016). This study was approved by the Research Ethics Committees of the respective hospitals, with the need for informed consent waived for this retrospective study. The exclusion criteria are presented in Additional file 1: Methods.

Clinicopathological characteristics information was collected, including age, sex, stage, tumor location, grade, and carcinoembryonic antigen (CEA) level. Stage was performed according to the Union for International Cancer Control guideline [20]. Preoperative CEA level was binarized as normal and abnormal groups, with the cut-off of 5 ng/mL. We focused on overall survival (OS), defined as the time from surgery to death for any reason.

### Whole-slide images acquisition and segmentation

**Fig. 1:**
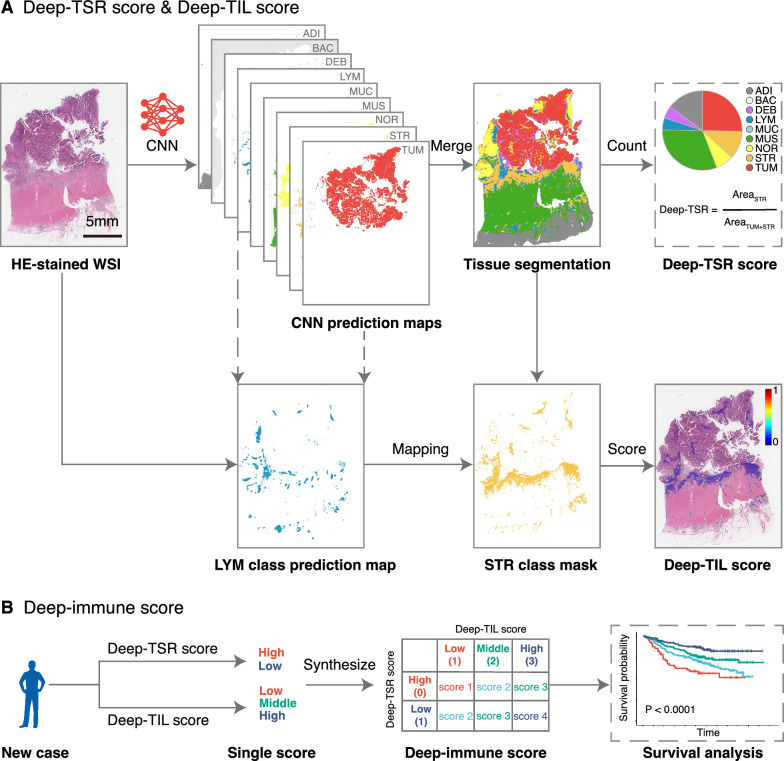
Study workflow. **A** Top panel: A CNN model (CNN-HE) was used to classify the HE-stained WSI of colorectal cancer into eight tissue types and one slide background. A rough segmentation map was obtained. The Deep-TSR score is calculated as "the area of STR /the area of STR and TUM". Bottom panel: Using STR of tissue segmentation as the mask, we define the Deep-TIL score as the mean prediction probability of LYM class in STR class. **B** The Deep-immune score was synthesized by the Deep-TSR score and Deep-TIL score. Deep-TSR-high and Deep-TSR-low groups were given 0 and 1 points, respectively. Deep-TIL-low, Deep-TIL-middle, and Deep-TIL-high groups were given 1, 2, and 3 points, respectively. A four-level scoring system (score 1–4) was established by summing both the Deep-TSR score and the Deep-TIL score. HE, hematoxylin and eosin; WSI, whole-slide image; CNN, convolutional neural network; ADI, adipose; BAC, background; DEB, debris; LYM, lymphocyte aggregates; MUC, mucus; MUS, muscle; NOR, normal mucosa; STR, stroma; TUM, tumor epithelium; TSR, tumor-stroma ratio; TIL, tumor-infiltrating lymphocyte

The HE-stained slide selection and digitization process are shown in Additional file 1: Methods. The stain type, stain location, and digital slide scanner for three different hospitals are presented in Additional file 1: Table S1. A convolutional neural network (CNN) model, VGG-19, was used to classify WSIs into eight tissue types and background. The detailed training process of the deep learning model (CNN-HE) has been reported in our previous work [16]. Then, HE-stained WSIs were tiled into overlapped patches (224 × 224 pixel^2^ at 20×). The model's prediction maps were reserved, and the tissue segmentation was obtained by setting each image patch as the tissue class with the max probability (Fig. 1A). The model training and segmentation were done in MATLAB environment (R2019a, MathWorks, USA). Additional file 1: Fig. S2 shows example of HE-stained WSIs and corresponding tissue segmentation results from three hospitals.

### Deep-TSR score and Deep-TIL score

With deep learning, CRC tissues within WSIs can be segmented into nine categories. We defined Deep-TSR score as the area of stroma divided by the area of stroma plus tumor epithelium. The calculation process of the Deep-TSR in HE-stained WSIs can be seen in Fig. 1A (top panel). Patients were divided into Deep-TSR-low and Deep-TSR-high groups using a cutoff of 50%.

We also noticed that the infiltration level of lymphocytes in stroma could be quantified by using the stroma region of tissue segmentation as the mask, combined with the prediction probability map of LYM class (Fig. 1A, bottom panel). We define the Deep-TIL score as the mean prediction probability of LYM class in the stroma. For each patient, a percentile value was calculated according to the distribution of the Deep-TIL score in the primary cohort. With 33% and 66% percentiles as thresholds, patients were classified into a 3-level group: low, middle, and high score.

### Deep-immune score

We further developed the Deep-immune score, synthesized by the Deep-TSR and Deep-TIL scores. The calculation details are as follows: Deep-TSR-high and Deep-TSR-low groups were given 0 and 1 points, respectively. Deep-TIL-low, Deep-TIL-middle, and Deep-TIL-high groups were given 1, 2, and 3 points, respectively. A four-level scoring system was established by summing both the Deep-TSR and Deep-TIL scores (Fig. 1B).

### Immunohistochemistry validation

We used a subgroup of patients for immunohistochemical validation to explore whether the Deep-immune score extracted from HE-stained WSIs could reflect the patient's immune status. We used the CD3^+^ T cells to measure immune infiltration in CRC. A consecutive section was processed for immunohistochemistry (Additional file 1: Fig. S1), and the details of the IHC (CD3^+^) staining procedure were presented in our previous work [19].

**Fig. 2:**
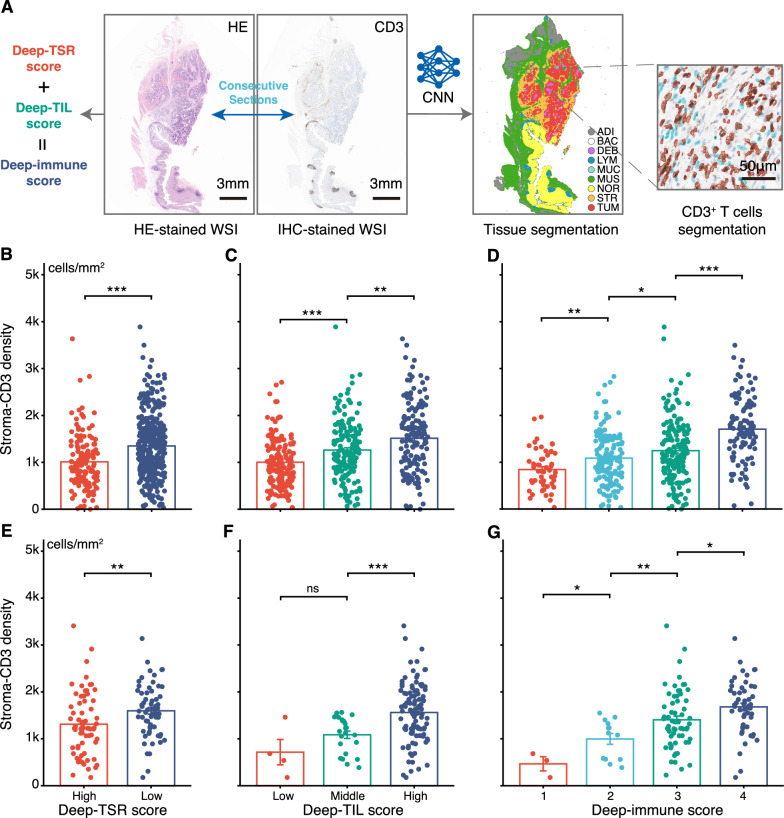
Association of Deep-TSR score, Deep-TIL score, and Deep-immune score with stroma-CD3 density. **A** A second CNN model (CNN-IHC) was used for tissue-level segmentation of IHC-stained WSI. The tissue types of the segmentation are the same as Fig. 1A. STR was used as the region of interest for WSI, and all CD3 + T-cells were segmented and counted within WSI. Then, the stroma-CD3 density was calculated by using the number of all CD3^+^ T cells divided by the STR area. **B-D** Student t-test was also used to compare the difference in stroma-CD3 density between groups with different scores (such as Deep-immune score 4 vs. 3) in primary cohort. **E–G** Student t-test was used in validation cohort to compare the difference in stroma-CD3 density between groups with different scores. (^ns^P > 0.05, *P < 0.05, **P < 0.01, ***P < 0.001, Student’s t-test). HE, hematoxylin and eosin; IHC, immunohistochemistry; WSI, whole-slide image; CNN, convolutional neural network; STR, stroma; TUM, tumor epithelium; TSR, tumor-stroma ratio; TIL, tumor-infiltrating lymphocyte

We used a second deep learning model (CNN-IHC), for tissue-level segmentation, to automatically obtain stroma regions in IHC-stained WSIs. The segmented stroma was used as the region of interest (ROI) for WSI, and all CD3^+^ T cells were segmented and counted using our previously developed software [21]. Then, the stroma-CD3 density was calculated by using the number of CD3^+^ T cells divided by the stroma area. The calculation process of the stroma-CD3 density is shown in Fig. 2A.

### Statistical analysis

Clinicopathological characteristics were compared by Student t-test for a continuous variable or Chi-square test for a category variable. Kaplan–Meier curves were plotted to determine difference in survival rates among groups, and log-rank tests were used to calculate P values. For multivariate analysis, univariate variables with P < 0.05 were selected. Hazard ratio (HR) with 95% confidence interval (CI) was calculated by using the Cox model. The discrimination performance of factor or model was assessed using Harrell's C-statistics (C-index) with 95% CI and the integrated area under the ROC curve (iAUC) of 0–5 years. All statistical analyses were conducted in R environment (version 4.0.3), with statistical significance set at 0.05.

## Results

### Patients

A total of 1010 CRC patients from three hospitals were included. Among them, 544 patients formed the primary cohort, with a median follow-up time for censored of 6.46 (interquartile range [IQR], 5.46–8.50) years; 466 patients formed the validation cohort, with a median follow-up time of 5.09 (IQR, 4.39–5.82) years. A comparison of clinicopathologic characteristics between the two cohorts is shown in Additional file 1: Table S2. For each patient, one HE-stained WSI was used to construct Deep-TSR, Deep-TIL, and Deep-immune scores. Among them, 477 from the primary cohort and 129 patients from the validation cohort had the paired IHC-stained WSIs for CD3^+^ T cells density evaluation (Additional file 1: Fig. S1).

### Association of Deep-TSR, Deep-TIL, and Deep-immune scores with stroma-CD3 density

For patients with IHC-stained WSIs in the primary cohort, 338 (71%) patients were grouped as Deep-TSR-low, and 139 (29%) patients were grouped as Deep-TSR-high. The distribution of stroma-CD3 density versus Deep-TSR is shown in Fig. 2B. Deep-TSR-low was associated with a high level of stroma-CD3 density (P < 0.001). For the Deep-TIL score, 164 (34%) cases were classified as a low score, 162 (34%) as middle score, and 151 (32%) as a high score. The mean stroma-CD3 density of the high-score group was 1.5 times higher than that of the low-score group (1513 vs. 1001 cells/mm^2^; P < 0.001; Fig. 2C). We also observed that with the increase in Deep-immune score, the mean density of the stroma-CD3 also increased (density in score 1–4: 844, 1088, 1246, and 1708 cells/mm^2^; Fig. 2D).

In the validation cohort, the Deep-TSR score, Deep-TIL score, and Deep-immune score had a similar trend to the primary cohort (Fig. 2E–G; Additional file 1: Table S3). Based on this analysis, high Deep-immune scores were associated with increasing levels of CD3^+^ T cells infiltration in the stroma region.

### Prognostic value of Deep-TIL score and Deep-TSR score

**Fig. 3:**
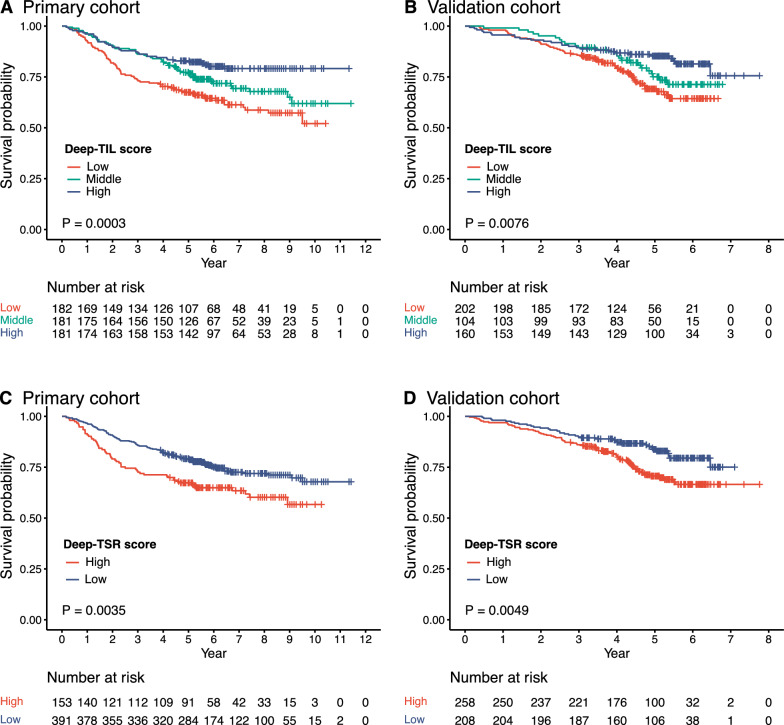
Kaplan–Meier plots for CRC patients according to Deep-TIL score and Deep-TSR score. **A** Deep-TIL score in the primary cohort; **B** Deep-TIL score in the validation cohort; **C** Deep-TSR score in the primary cohort; **D** Deep-TSR score in the validation cohort. TSR, tumor-stroma ratio; TIL, tumor-infiltrating lymphocyte

In the primary cohort, overall survival was significantly longer for patients with higher Deep-TIL scores. The 5-year survival rates were 67.4% in Deep-TIL-low group, 76.6% in middle group, and 82.9% in high group (P = 0.0003; Fig. 3A). Patients with high and low Deep-TIL scores experienced a significant difference in survival (unadjusted HR 0.45, 95% CI 0.30 − 0.67; P < 0.001; Table 1). In the validation cohort, these findings were confirmed (0.49, 0.31–0.77; 0.002; Fig. 3B; Table 1).

**Table 1:** Uni– and multivariate analyses including TNM, sex, age, location, CEA, grade, Deep-TSR score, Deep-TIL score, and Deep-immune score for OS in the two cohorts

	Univariate analysis				Multivariate analysis			
	Primary cohort		Validation cohort		Primary cohort		Validation cohort	
	HR (95% CI)	P	HR (95% CI)	P	HR (95% CI)	P	HR (95% CI)	P
*TNM*								
I	1		1		1		1	
II	2.48 (1.12–5.50)	0.025	1.53 (0.54–4.34)	0.400	2.56 (1.01–6.51)	0.048	1.14 (0.39–3.36)	0.800
III	6.17 (2.87–13.3)	< 0.001	3.94 (1.44–10.8)	0.008	5.78 (2.33–14.4)	< 0.001	2.70 (0.94–7.79)	0.066
*Sex*								
Male	1		1					
Female	1.05 (0.71–1.56)	0.800	0.78 (0.33–1.84)	0.600				
Age	1.03 (1.01–1.04)	< 0.001	1.03 (1.01–1.04)	0.001	1.03 (1.01–1.04)	< 0.001	1.03 (1.01–1.04)	< 0.001
*Location*								
Colon	1		1					
Rectum	1.00 (0.73–1.37)	0.999	1.48 (1.00–2.21)	0.053				
*CEA*								
Normal	1		1		1		1	
Abnormal	2.58 (1.87–3.56)	< 0.001	1.98 (1.35–2.91)	< 0.001	1.94 (1.40–2.70)	< 0.001	1.46 (0.46–1.18)	0.200
*Grade*								
Low	1		1					
High	1.44 (0.91–2.28)	0.120	1.80 (1.22–2.67)	0.003				
*Deep-TSR score*								
High	1		1					
Low	0.62 (0.45–0.86)	0.004	0.57 (0.38–0.85)	0.005				
*Deep-TIL score*								
Low	1		1					
Middle	0.69 (0.48–0.99)	0.044	0.73 (0.46–1.17)	0.200				
High	0.45 (0.30–0.67)	< 0.001	0.49 (0.31–0.77)	0.002				
*Deep-immune score*								
1	1		1		1		1	
2	0.68 (0.43–1.08)	0.100	0.72 (0.45–1.14)	0.200	0.67 (0.42–1.10)	0.110	0.73 (0.46–1.18)	0.200
3	0.48 (0.30–0.77)	0.002	0.48 (0.29–0.79)	0.004	0.54 (0.33–0.90)	0.019	0.64 (0.38–1.07)	0.091
4	0.27 (0.15–0.48)	< 0.001	0.31 (0.15–0.62)	< 0.001	0.36 (0.20–0.66)	0.001	0.41 (0.20–0.84)	0.016

TNM, tumor-node-metastasis; CEA, carcinoembryonic antigen; TSR, tumor-stroma ratio; TIL, tumor-infiltrating lymphocytes; OS, overall survival; HR, Hazard ratio; CI, confidence interval

In the primary cohort, the 5-year overall survival for CRC patients with low and high Deep-TSR scores were 78.9% and 67.3% respectively (unadjusted HR for low vs. high 0.62, 95% CI 0.45–0.86; P = 0.004; Fig. 3C). Validation cohort results confirm the initial findings: the 5-year survival rates for low-stroma and high-stroma patients were 82.7% and 72.2%, respectively, with unadjusted HR (low vs. high) of 0.57 (0.38–0.85; 0.005; Fig. 3D).

### Prognostic value of Deep-immune score

**Fig. 4:**
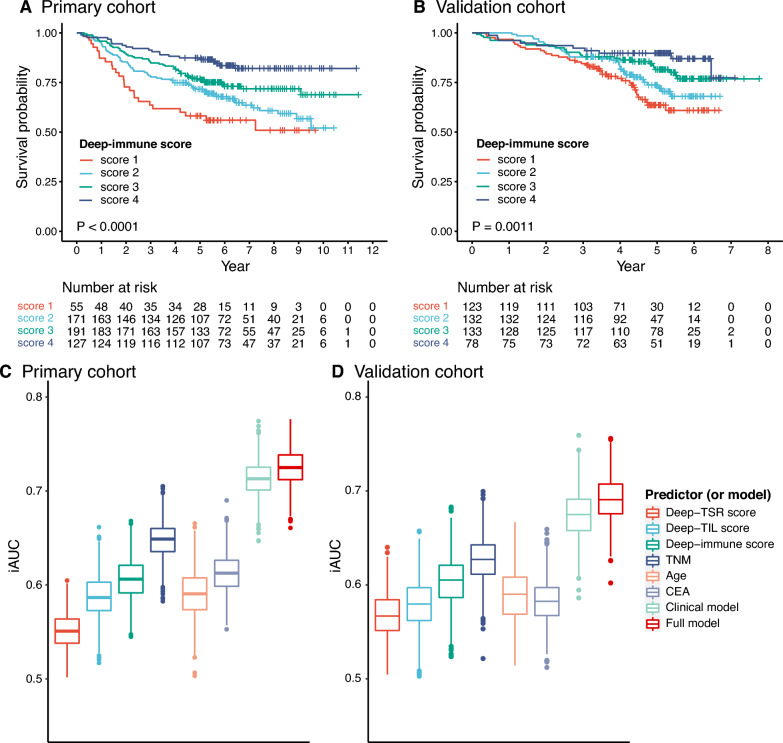
Kaplan–Meier and iAUC plots. Kaplan–Meier plots of Deep-immune score in primary cohort (**A**) and in the validation cohort (**B**). The iAUC of 0–5 years of factors and models in primary cohort (**C**) and in the validation cohort (**D**). TSR, tumor-stroma ratio; TIL, tumor-infiltrating lymphocytes. TNM, tumor-node-metastasis; CEA, carcinoembryonic antigen; iAUC, the integrated area under the ROC curve

The Deep-immune score was synthesized by the Deep-TSR score and the Deep-TIL score. In the primary cohort, a significant difference was observed in 5-year survival rates between score 4 and 1 groups: 87.4% vs. 58.2% (P < 0.001; Fig. 4A). Patients with the highest score had the most favorable OS (unadjusted HR for score 4 vs. score 1 0.27, 95% CI 0.15–0.48; P < 0.001; Table 1).

Similar trends were observed in the validation cohort. Compared with score 1, patients with score 4 of Deep-immune score had favorable outcomes (unadjusted HR for score 4 vs. score 1 0.31, 95% CI 0.15–0.62, P < 0.001; Table 1). The 5-year survival rates of score 4 and score 1 groups were 89.8% and 67.0%, respectively (Fig. 4B). The results showed that a combined analysis of two scores proved to be more accurate at predicting outcomes.

### Survival analysis of scores stratified with stage

In addition, when we examined whether Deep-TSR score could be applied to subgroups of patients with stage, based on stratification by stage I and II, the score had no statistically significant impact on the prognosis. (All P > 0.05; Additional file 1: Fig. S3A, C, E). As the Deep-TIL score, only a marginally statistically significance was found among stage II individuals (P = 0.051; Additional file 1: Fig. S3F). However, the Deep-immune score showed good prognostic ability in all stage I–III subgroups (All P < 0.05; Additional file 1: Fig. S4), especially in the stage II group (P = 0.018).

### The added value of the Deep-immune score

In Table 1, the univariate association between clinicopathological characteristics and OS is presented. We identified age, stage, CEA level, Deep-TSR score, Deep-TIL score, and Deep-immune score as prognostic factors for OS (P < 0.05). In multivariate analysis, Deep-immune score was still associated with OS, independent of age, CEA level, and stage. There was an association between a lower Deep-immune score and a shorter OS in the primary (adjusted HR for score 4 vs. score 1 0.36, 95% CI 0.20–0.66, P = 0.001) and validation cohorts (0.41, 0.20–0.84, 0.016; Table 1).

Two Cox models were developed in order to evaluate the added prognostic value of the Deep immune score: the full model incorporated the independent predictors (stage, age, CEA level, and Deep-immune score) in multivariate analysis of the primary cohort, and a clinical model incorporated clinical factors. The model with the Deep-immune score had a better discrimination performance than the clinical model (C-index: 0.732 vs. 0.720; iAUC: 0.726 vs. 0.713; Fig. 4C). The results of the validation cohort were similar as well (0.701 vs. 0.689; 0.691 vs. 0.676; Additional file 1: Table S4, Fig. 4D).

## Discussion

The AI-based method could quantify the tissue composition in TME with HE-stained WSIs of CRC. To understand the basis of tumor heterogeneous clinical behavior, many scholars have focused on TME [22, 23]. Studies have shown that TME characterization provides additional insight into the prognosis of patients with solid tumors [24, 25]. The stroma of TME was the focus of studying the prognosis of CRC. Our previous analysis and studies by other scholars have shown that in CRC patients, abundant stroma in tumor tissue was associated with poor prognosis [16, 26]. Results of our present work also suggested that stroma proportion quantified in HE-stained WSIs can help stratify risk of CRC patients. Patients who have Deep-TSR-high scores have a much lower 5-year survival rate than those who have low scores (67.3% vs. 78.9%). Remodeling of stroma can serve as a physical barrier to prevent tumor cells from coming into contact with immune cells [27–29]. The mean stroma-CD3 density in the Deep-TSR-low group was 1350 cells/mm^2^, higher than that in the Deep-TSR-high group (1011 cells/mm^2^). Additionally, the stroma contains special connective tissues such as fibroblasts, mesenchymal stromal cells, osteoblasts and chondrocytes, along with extracellular matrix. The endothelial cells within it provide nourishment for tumor growth, constitute a pathway for metastatic spread through angiogenesis, and lead to resistance to chemotherapy and radiation therapy [23, 30, 31]. Therefore, the more stromal components, the lower the OS of patients.

Furthermore, the stroma was incredibly intricate. For example, desmoplastic reaction was classified as immature, intermediate, or mature according to the different connective tissue-promoting reactions in the stroma [32]. Moreover, according to cancer-immune phenotypes, anticancer immunity in humans can be categorized into three main types: the immune-desert, the immune–excluded, and the inflamed phenotypes [33]. Studies have shown that the content and density of TILs in the stroma were also attached to OS [15, 34]. A patch-level segmentation was performed in our work without dissecting each lymphocyte with precision, which cannot accurately quantify the density and spatial location of lymphocytes. However, we noted that the class of lymphocytes, one of the tissue categories in our model, were structured made up of clusters of TILs. Therefore, we tried to take the result of stroma segmentation as ROI and defined the mean predictive probability of the ROI for this category of LYM as the Deep-TIL score. Besides, we found that the mean stroma-CD3 density of the high-score group was 1.5 times higher than the low group. The automatic quantification of the Deep-TIL score could reflect the immune cells infiltration in the stroma region. Survival analysis showed that the Deep-TIL score could stratify the prognosis of CRC. The higher the Deep-TIL score, the longer the survival time. The 5-year survival rate was recorded for 70.2% of patients with a low score, 75.7% of patients with a middle score, and 85.4% of patients with a high score. This scoring method was kind to use, and this method only needed the label of the patch, which was less computationally intensive. More worth mentioning was that it also took into account the spatial distribution of TILs in the stroma.

Tumor growth pattern, aggressiveness, metastasis, and patient prognosis are the result of a combination of multiple factors. These include the interaction between components of TME from the cellular level to the tissue level [35, 36]. Based on this, after completing the above two scores, the Deep-TSR score, and Deep-TIL score, we raised the conjecture whether the combination of the two scores could reflect more prognostic information. Patients with the highest score had the most favorable OS (unadjusted HR for score 4 vs. score 1: 0.27). Similar results were also found in the validation cohort, which revealed that our score was robust. Furthermore, we found that the full model, including Deep-immune score and clinicopathological factors, had a higher prognostic value than a clinicopathological model (iAUC, 0.726 vs. 0.713). Combined with clinicopathological factors, the prognosis of patients with CRC could be evaluated in a more comprehensive and integrated manner. We also observed that with the increase in the Deep-immune score, the stroma-CD3 density also increased. CD3^+^ T cells are membrane markers of mature T lymphocytes that can be used to quantify the total number of T lymphocytes [19]. When CD3^+^ cells was increased, it represented a higher tumor lymphatic infiltration and a higher amount of tumor-killing immune cells, which has a protective effect on the organism [37, 38]. This result supports the idea that our proposed Deep-immune score may be sufficient to predict prognosis of CRC. In addition, the Deep-immune score, which was fully automated and with HE-stained as a routine staining method and IHC-stained as a special staining, has certain economic benefits. There have been many studies suggesting that TSR and TILs could predict prognosis of other solid tumors. Take breast cancer as an example. Studies on TSR found a significant association between high tumor stroma content and poor prognosis [39, 40]. The results of related studies on TILs showed that increased TILs concentrations were associated with increased frequency of adjuvant chemotherapy responses in all breast cancer subtypes, and they were also associated with longer survival in patients with triple-negative breast cancer and HER2-positive breast cancer [41, 42]. The results were similar to those of our study in CRC. This suggested that if we had segmented breast cancer tissues and defined the corresponding types of tissues, using our method to calculate tumor-stroma ratio, Deep-TIL score, and Deep-immune score, could also predict prognosis for breast cancer patients. Therefore, our method has the potential ability to be applied to other solid tumors.

In stage II patients, neither Deep-TSR nor Deep-TIL score can distinguish between high-risk and low-risk CRC individuals (all P > 0.05). However, the composite score can stratify patients' prognostic risk (P = 0.018). This indicated that Deep-immune score had the potential to guide clinical risk stratification of patients with stage II CRC, which in turn could influence clinical decision-making.

## Conclusions

In conclusion, we proposed a Deep-immune score that can fully quantify the HE-stained WSI of CRC by artificial intelligence. Evidence suggests that a Deep-immune score may reflect the immune state of CRC patients and be associated with better survival. Findings based on digital pathology could be particularly useful for adjusting risk stratification of CRC and impacting subsequent precision medicine.

## Supplementary Information

**Additional file 1.**

## Data Availability

The data sets for training the model were available online (https://doi.org/10.5281/zenodo.4024676, https://doi.org/10.5281/zenodo.4023999).

## Abbreviations

TME: Tumor microenvironment
CRC: Colorectal cancer
TNM: Tumor-node-metastasis
TIME: Tumor immune microenvironment
TILs: Tumor-infiltrating lymphocytes
IHC: Immunohistochemistry
HE: Hematoxylin and eosin
WSIs: Whole-slide images
CLR: Crohn-like lymphoid reaction
TSR: Tumor-stroma ratio
AI: Artificial intelligence
CEA: Carcinoembryonic antigen
OS: Overall survival
CNN: Convolutional neural network
ROI: Region of interest
HR: Hazard ratio
CI: Confidence intervals
iAUC: Integrated area under the ROC curve
IQR: Interquartile range
