# Correction: Artificial intelligence for quantifying immune infiltrates interacting with stroma in colorectal cancer

**DOI:** 10.1186/s12967-024-05968-0

**Published:** 2025-01-30

**Authors:** Jing Yang, Huifen Ye, Xinjuan Fan, Yajun Li, Xiaomei Wu, Minning Zhao, Qingru Hu, Yunrui Ye, Lin Wu, Zhenhui Li, Xueli Zhang, Changhong Liang, Yingyi Wang, Yao Xu, Qian Li, Su Yao, Dingyun You, Ke Zhao, Zaiyi Liu

**Affiliations:** 1 Guangdong Provincial Key Laboratory of Artificial Intelligence in Medical Image Analysis and Application, Guangdong Provincial People’s Hospital, Guangzhou, China; 2 Department of Cardiology, Sun Yat-Sen Memorial Hospital, Sun Yat-Sen University, Guangzhou, China; 3 Department of Radiology, Guangdong Provincial People’s Hospital, Guangdong Academy of Medical Sciences, 106 Zhongshan Er Road, Guangzhou, 510080 China; 4 The Second School of Clinical Medicine, Southern Medical University, Guangzhou, China; 5 Department of Pathology, The Sixth Affiliated Hospital of Sun Yat-Sen University, Guangzhou, China; 6 Department of Radiology, The Sixth Affiliated Hospital of Sun Yat-Sen University, Guangzhou, China; 7 Department of Pathology, The Third Affiliated Hospital of Kunming Medical University, Yunnan Cancer Hospital, Yunnan Cancer Center, Kunming, China; 8 Department of Radiology, The Third Affiliated Hospital of Kunming Medical University, Yunnan Cancer Hospital, Yunnan Cancer Center, Kunming, China; 9 Department of Ophthalmology, Guangdong Eye Institute, Guangdong Provincial People’s Hospital, Guangdong Academy of Medical Sciences, Guangzhou, China; 10 Department of Radiology, Zhuhai People’s Hospital, Zhuhai Hospital Affiliated With Jinan University, Zhuhai, China; 11 School of Medicine, South China University of Technology, Guangzhou, China; 12 Department of Pathology, Guangdong Provincial People’s Hospital, Guangdong Academy of Medical Sciences, 106 Zhongshan Er Road, Guangzhou, 510080 China; 13 School of Public Health, Kunming Medical University, 191 West Renmin Road, Kunming, 650500 China; 14 Guangdong Cardiovascular Institute, Guangdong Provincial People’s Hospital, Guangdong Academy of Medical Sciences, Guangzhou, China

**Correction: Journal of Translational Medicine (2022) 20:451**

10.1186/s12967-022-03666-3

The authors of the original article [1] have found out after publication that there was an error in Fig. 4C. The correct (Fig. 1) and incorrect figure (Fig. 2) are shown in this correction article.

It is now as follows:

**Fig. 1:**
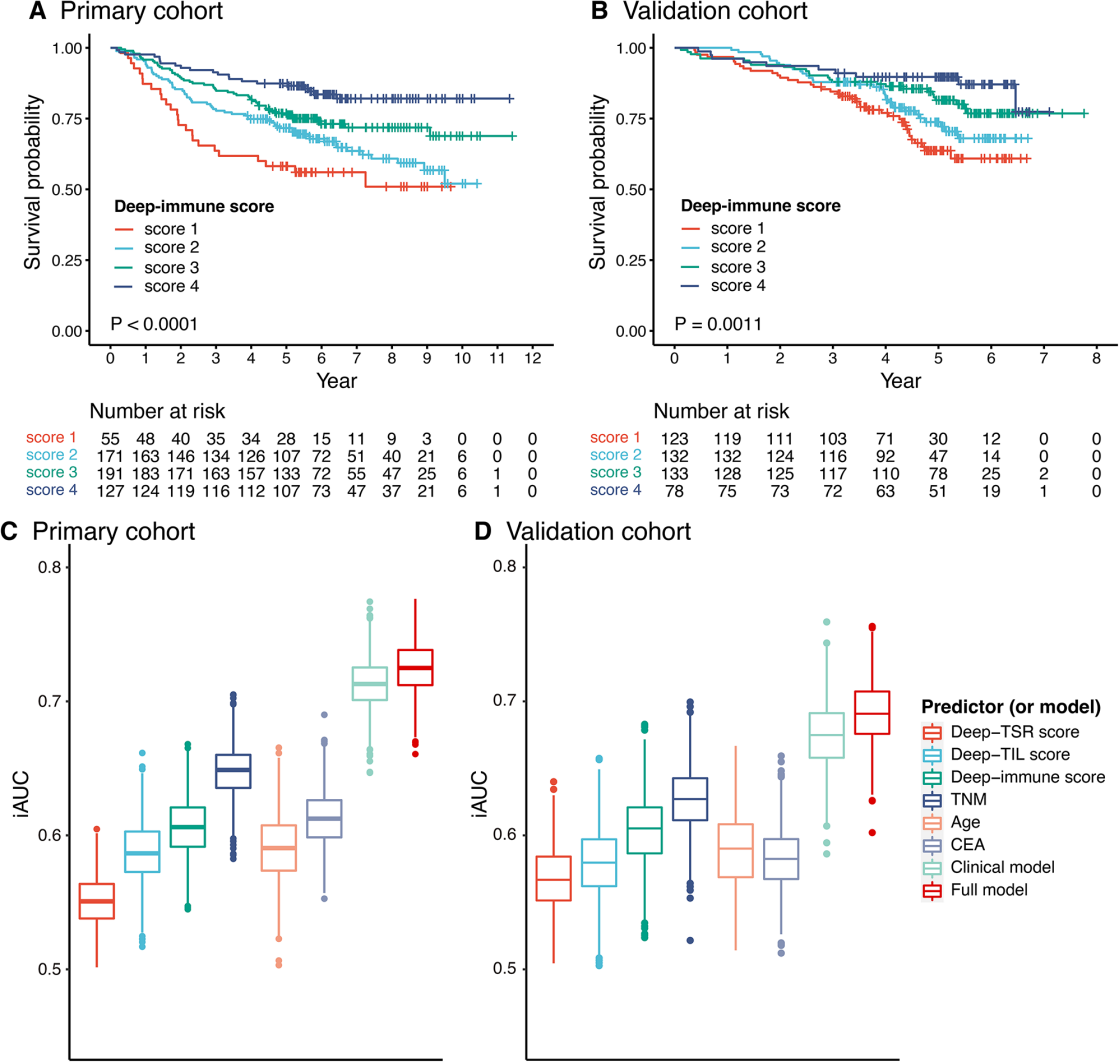
Kaplan–Meier and iAUC plots. Kaplan–Meier plots of Deep-immune score in primary cohort (**A**) and in the validation cohort (**B**). The iAUC of 0–5 years of factors and models in primary cohort (**C**) and in the validation cohort (**D**). TSR, tumor-stroma ratio; TIL, tumor-infiltrating lymphocytes; TNM, tumor-node-metastasis; CEA, carcinoembryonic antigen; iAUC, the integrated area under the ROC curve

It should be as follows:

**Fig. 2:**
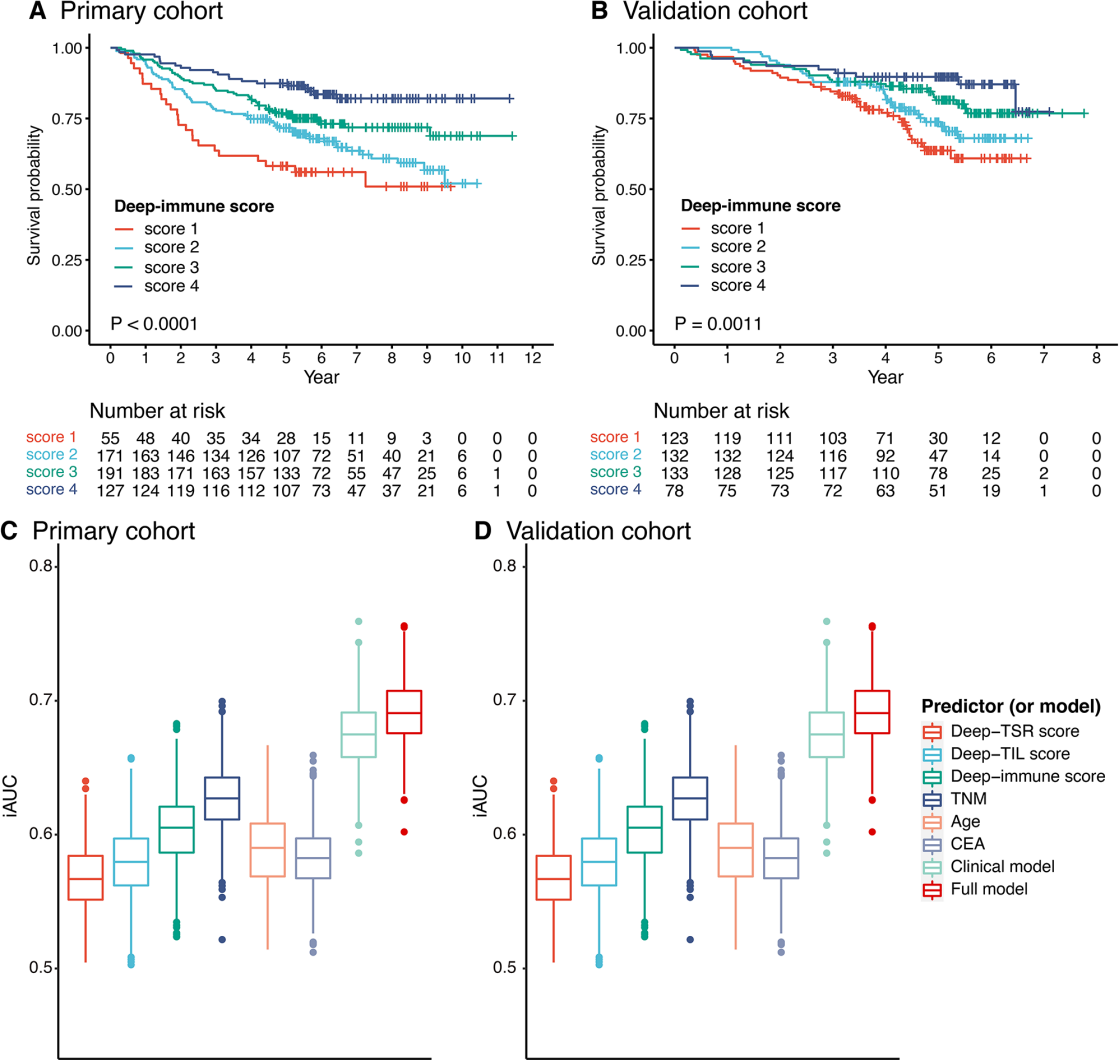
Kaplan–Meier and iAUC plots. Kaplan–Meier plots of Deep-immune score in primary cohort (**A**) and in the validation cohort (**B**). The iAUC of 0–5 years of factors and models in primary cohort (**C**) and in the validation cohort (**D**). TSR, tumor-stroma ratio; TIL, tumor-infiltrating lymphocytes; TNM, tumor-node-metastasis; CEA, carcinoembryonic antigen; iAUC, the integrated area under the ROC curve

This correction does not affect either results or conclusions. We apologize for the inconvenience to the readers.
