# Supplementary material for: Artificial intelligence for quantifying immune infiltrates interacting with stroma in colorectal cancer
Source: J Transl Med. 2022 Oct 4;20:451. doi: 10.1186/s12967-022-03666-3 (PMC9533523; doi:10.1186/s12967-022-03666-3)
Supplement: Supplementary file 1 — Additional file 1. [file 12967_2022_3666_MOESM1_ESM.pdf]

Additional file for

Artificial intelligence for quantifying immune infiltrates interacting with stroma in colorectal cancer

This PDF file includes:

Additional Methods

Additional Figures (S1–S4)

Additional Tables (S1–S4)

Additional Methods

Exclusion criteria were as follows

(1) Neo-adjuvant therapy (radiotherapy, chemotherapy); (2) Death within 30 days of surgery; (3) Follow-up information missing; (4) Hematoxylin and eosin-stained whole-slide images unavailable or poor image quality.

Whole-slide images acquisition

Pathologists from three centers selected one tumor block from each patient that contained the most invasive part of the primary tumor. One tissue paraffin section of 4–5 μm was stained with hematoxylin and eosin for all patients. The slides were digitized using digital slide scanner (Aperio AT2 or GT 450, Leica, USA; KF-PRO-020, KFBIO, China) at 40× magnification (resolution: 0.24–0.26 μm/pixel).

Additional Figures

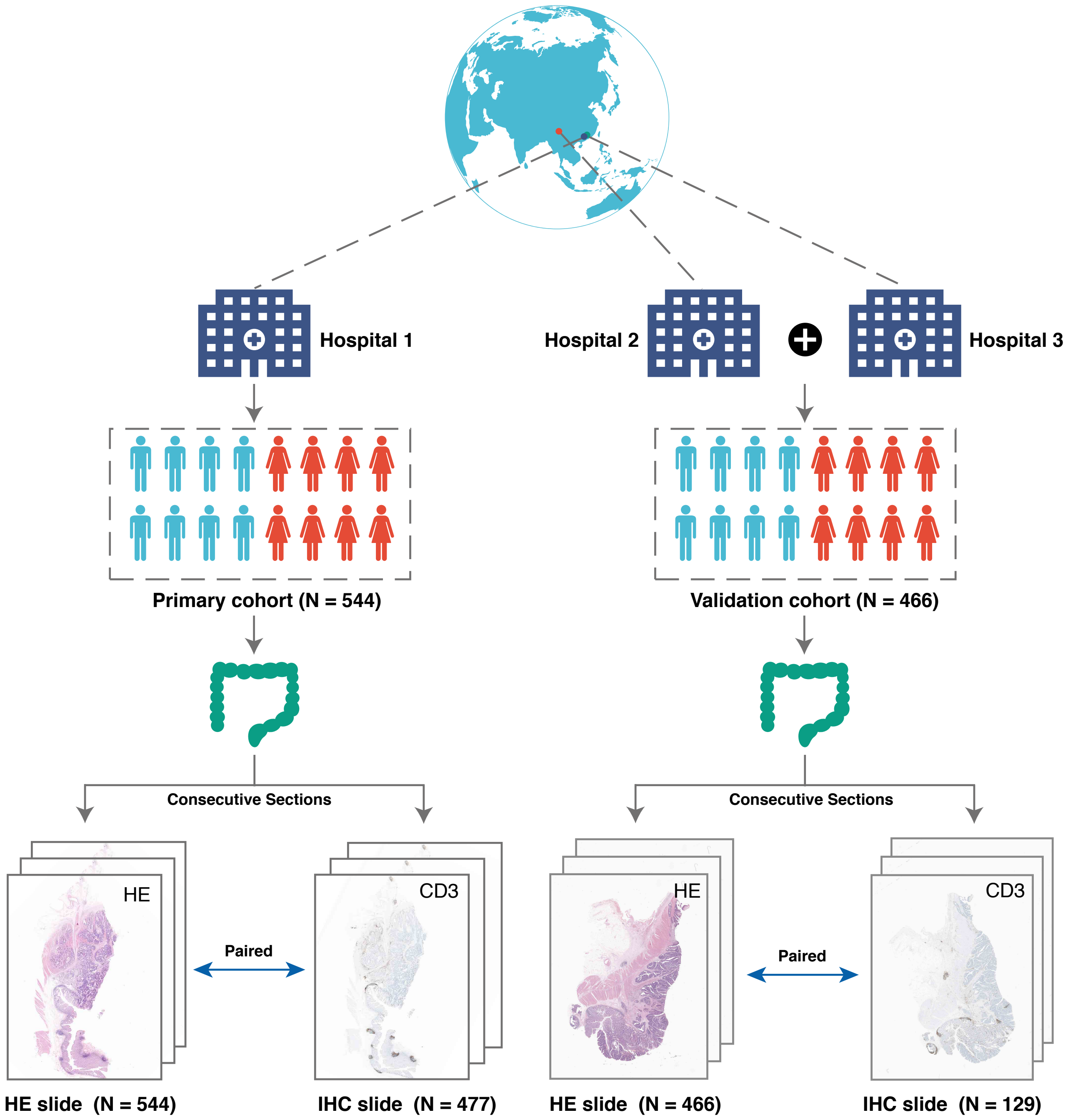

Fig. S1 Study profile. The primary cohort (N = 544) consists of colorectal cancer (CRC) patients from Hospital 1, and the validation cohort (N = 466) includes patients from Hospital 2 and Hospital 3. One tissue paraffin section of 4–5 μm was stained with hematoxylin and eosin (HE) for all patients (N = 544 in the primary cohort, N = 466 in the validation cohort). And the consecutive sections were processed for immunohistochemistry (IHC) for a subgroup of all patients (N = 477 in the primary cohort, N = 129 in the validation cohort).

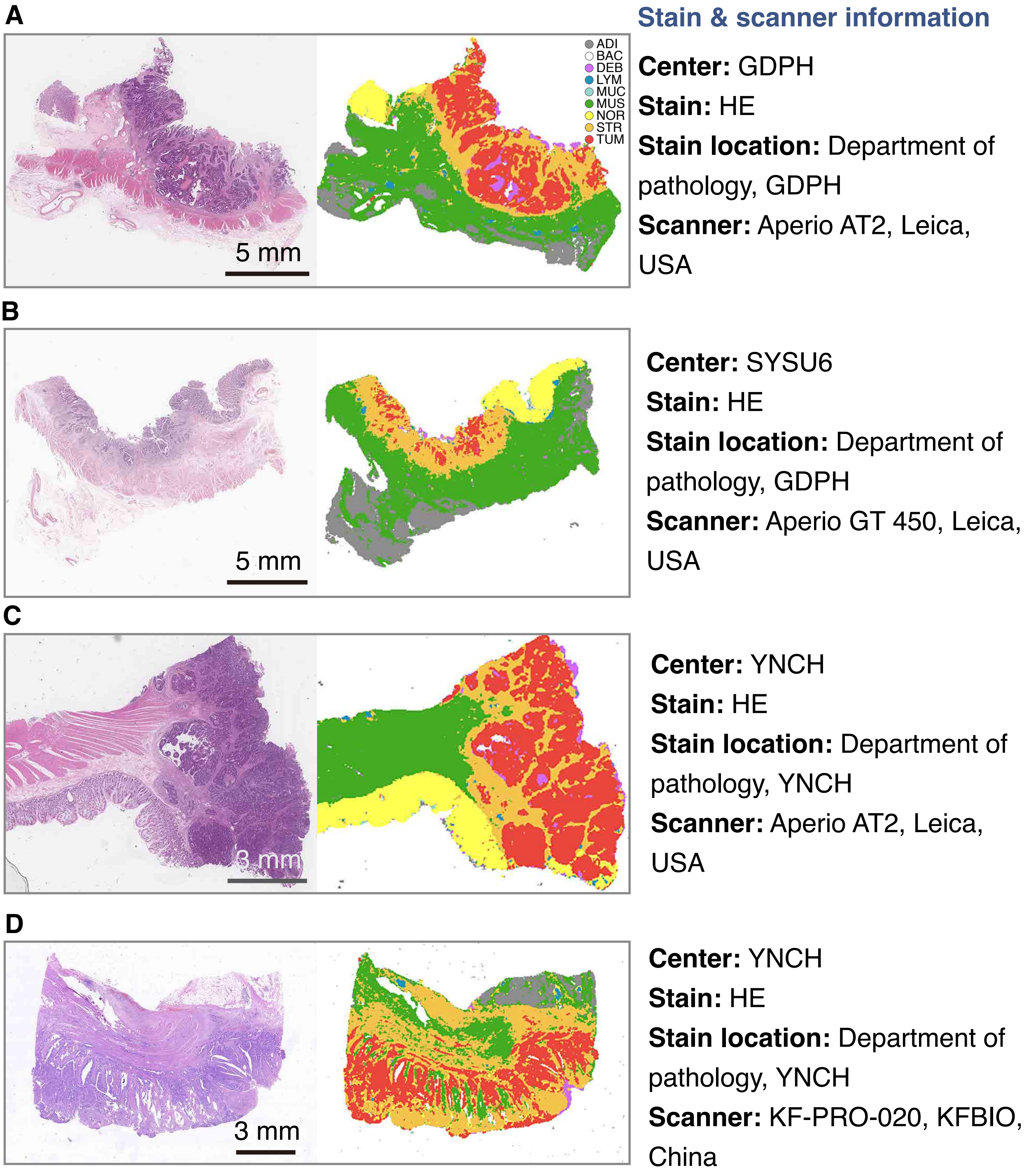

Fig. S2 HE-stained WSIs and tissue segmentation from three hospitals. GDPH, Guangdong Provincial People's Hospita l; YNCH, Yunnan Cancer Hospital; SYSU6, The Sixth Affiliated Hospital of Sun Yat-sen University; HE, hematoxylin and eosin; WSI, whole-slide image.

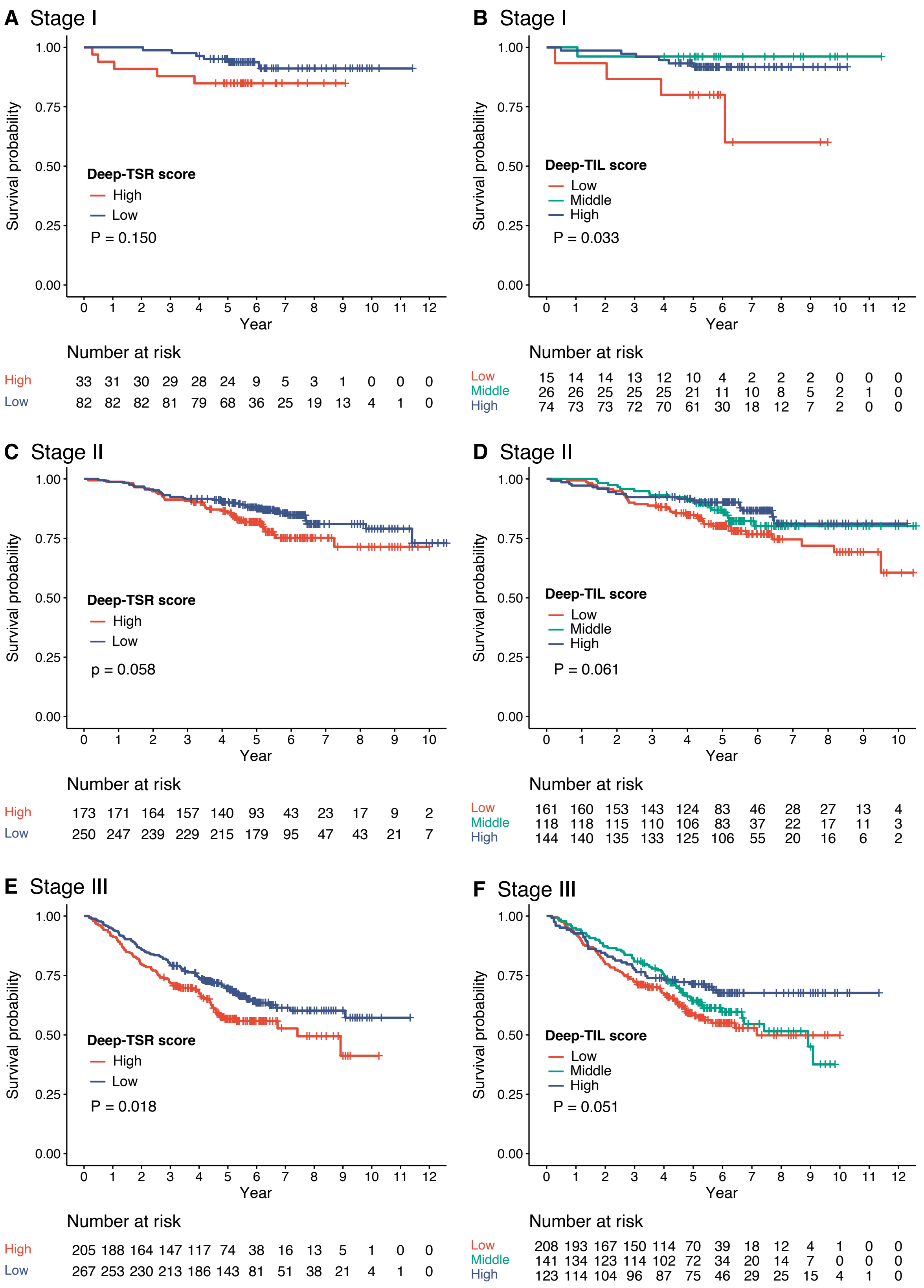

Fig. S3 Kaplan–Meier plots for all patients according to Deep-TSR and Deep-TIL scores, stratified by TNM stage I–III. TSR, tumor-stroma ratio; TIL, tumor-infiltrating lymphocyte; TNM, tumor-node-metastasis.

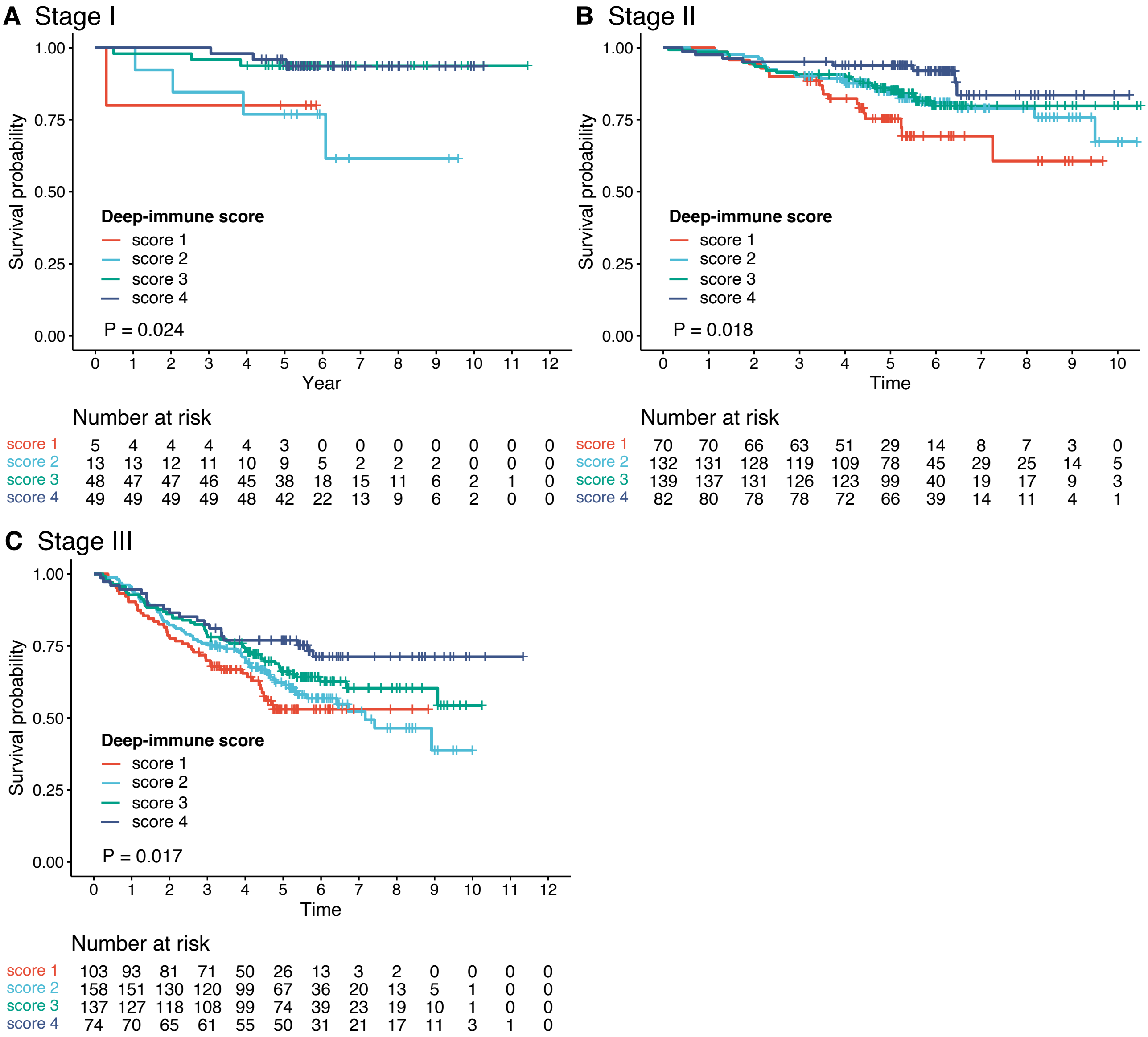

Fig. S4 Kaplan–Meier plots for all patients according to Deep-immune score, stratified by TNM stage. TNM, tumor-node-metastasis.

Additional Tables

Table S1. Stain type, stain location, and digital slide scanner for 3 different hospitals

Center	Stain type	Stain location	Digital slide scanner
GDPH (Hospital 1)	HE & IHC	Department of pathology, GDPH	Aperio AT2, Leica, USA
YNCH (Hospital 2)	HE	Department of pathology, YNCH	Aperio AT2, Leica, USA & KF-PRO-020, KFBIO, China
SYSU6 (Hospital 3)	HE & IHC	Department of pathology, GDPH	Aperio GT 450, Leica, USA

Abbreviations: GDPH, Guangdong Provincial People's Hospital; YNCH, Yunnan Cancer Hospital; SYSU6, The Sixth Affiliated Hospital of Sun Yat-sen University; IHC, immunohistochemistry; HE, hematoxylin and eosin.

Table S2. The distributions of demographic and clinicopathologic characteristics of colorectal cancer patients in the two cohorts

	Primary cohort	Validation cohort	P
**Age (year, mean ± SD)**	63.37 ± 12.24	59.15 ± 13.07	<0.001*
**Sex**			1#
Male	325 (59.7%)	279 (59.9%)	
Female	219 (40.3%)	187(40.1%)	
**TNM**			0.003#
I	79 (14.5%)	36 (7.7%)	
II	221 (40.6%)	202 (43.3%)	
III	244 (44.9%)	228(48.9%)	
**Location**			<0.001#
Colon	312 (57.4%)	170 (36.5%)	
Rectum	232 (42.6%)	296 (63.5%)	
**CEA**			<0.001#
Normal	331 (60.8%)	257 (55.2%)	
Abnormal	166 (30.5%)	204 (43.8%)	
NA	47 (8.6%)	5 (1.1%)	
**Grade**			<0.001#
Low	487 (89.5%)	300 (64.4%)	
High	57 (10.5%)	149 (32.0%)	
NA	0 (0%)	17 (3.6%)	

Note: P-value was performed by t-test or χ^2 test where appropriate. (* t-test; # Chi-square test)

Abbreviation: SD, standard deviation; TNM, tumor-node-metastasis; CEA, carcinoembryonic antigen.

Table S3. $CD3^+$ T cells density in the stroma region stratified by Deep-TSR, Deep-TIL, and Deep-immune scores

		Stroma-CD3+ T cells density (mean ± SD, cells/mm^2)	
		Primary cohort	Validation cohort
**Deep-TSR score**	Low	1350.13 ± 677.56	1596.31 ± 557.81
	High	1011.41 ± 582.04	1310.81 ± 665.56
**Deep-TIL score**	Low	1001.04 ± 518.93	714.49 ± 541.38
	Middle	1261.35 ± 635.78	1085.43 ± 381.10
	High	1512.73 ± 744.68	1561.36 ± 630.51
**Deep-immune score**	1	844.21 ± 429.48	465.69 ± 261.19
	2	1087.60 ± 548.92	996.95 ± 421.80
	3	1246.27 ± 666.92	1404.56 ± 621.30
	4	1705.75 ± 690.32	1679.24 ± 574.65

Abbreviation: TSR, tumor-stroma ratio; TIL, tumor-infiltrating lymphocyte; SD, standard deviation.

Table S4. The prediction performance in two cohorts

	Primary cohort		Validation cohort	
	**C-index (95% CI)**	**iAUC**	**C-index (95% CI)**	**iAUC**
**Deep-TSR score**	0.557 (0.519–0.596)	0.550	0.568 (0.521–0.615)	0.567
**Deep-TIL score**	0.583 (0.539–0.627)	0.587	0.576 (0.523–0.629)	0.580
**Deep-immune score**	0.605 (0.562–0.648)	0.606	0.602 (0.549–0.656)	0.605
**TNM stage**	0.653 (0.617–0.690)	0.647	0.625 (0.579–0.672)	0.626
**Age**	0.588 (0.541–0.636)	0.591	0.601 (0.546–0.657)	0.589
**CEA**	0.626 (0.586–0.666)	0.611	0.600 (0.552–0.647)	0.583
**TNM + Age + CEA**	0.720 (0.682–0.759)	0.713	0.689 (0.641–0.738)	0.676
**Deep-immune score + TNM + Age + CEA**	0.732 (0.695–0.770)	0.726	0.701 (0.652–0.751)	0.691

Abbreviations: CI, confidence interval; iAUC, the integrated area under the ROC curve; TSR, tumor-stroma ratio; TIL, tumor-infiltrating lymphocytes; TNM, tumor-node-metastasis; CEA, carcinoembryonic antigen.
